# Multivariate Statistical Analysis for the Classification of Sausages Based on Physicochemical Attributes, Using Attenuated Total Reflectance-Fourier Transform Infrared (ATR-FTIR) and Inductively Coupled Plasma-Mass Spectrometry (ICP-MS)

**DOI:** 10.1155/2024/1329212

**Published:** 2024-03-12

**Authors:** Quang Minh Bui, Quang Trung Nguyen, Thanh Thao Nguyen, Ha My Nguyen, Thi Tinh Phung, Viet Anh Le, Ngoc Minh Truong, The Vinh Mac, Tien Dat Nguyen, Le Tuan Anh Hoang, Ha Minh Duc Tran, Van Nhan Le, Minh Duc Nguyen

**Affiliations:** ^1^Center for High Technology Research and Development (CHTD), Vietnam Academy of Science and Technology (VAST), 18 Hoang Quoc Viet Road, Hanoi, Vietnam; ^2^Institute of Environmental Science and Public Health, Ho Chi Minh City, Vietnam; ^3^Institute of Environmental Technology, Vietnam Academy of Science and Technology (VAST), 18 Hoang Quoc Viet Road, Hanoi, Vietnam; ^4^Hanoi University of Industry, 298 Cau Dien Street, Hanoi, Vietnam; ^5^Institute of Genome Research, Vietnam Academy of Science and Technology (VAST), 18 Hoang Quoc Viet Road, Hanoi, Vietnam

## Abstract

Sausage is a convenient food that is widely consumed in the world and in Vietnam. Due to the rapid development of this product, the authenticity of many famous brands has faded by the rise of adulteration. Therefore, in this study, principal component analysis (PCA) was combined with chemical analysis to identify 6 sausage brands. Sausage samples were dried and then ground to a fine powder for both instrumental analyses of attenuated total reflectance-Fourier transform infrared (ATR-FTIR) and inductively coupled plasma–mass spectrometry (ICP-MS). Dried measurements of ATR-FTIR was performed directly on the ZnSe crystal, while elemental data were obtained through microwave digestion before the ICP-MS analysis. Principal component analysis (PCA) within the framework software of XLSTAT and STATISTICA 12 was performed on the spectroscopy and elemental dataset of sausage samples. PCA visualized the distinction of 6 sausage brands on both datasets of ATR-FTIR and ICP-MS. The classification on the spectroscopy profile showed that although more than 90% variation of the dataset was explained on the first two PCs, the difference between several brands was not detected as the distribution of data overlapped with one another. The PCA observation of the elemental composition on PC1 and PC3 has separated the sausage brands into 6 distinctive groups. Besides, several key elements contributed to the brands' identification have been detected, and the most distinctive elements are Na, K, Ca, and Ba. PCA visualization showed the feasibility of the classification of sausage samples from different brands when combined with the results of FT-IR and ICP-MS methods. The experiment was able to differentiate the sausages from the 5 brands using multivariate statistics.

## 1. Introduction

Sausage is processed food with the main ingredients consisting of types of meat (pork, beef, and chicken), salt, herbs, and spices [1, 2]. According to data from Statista [3], the top market for meat and sausages in 2022 is the United States at the top (38,976.6 million USD), Germany second (33,611.68 million USD), and France at third (17,264.51 million USD). With many advantages of high nutrition and convenient usage, sausage is favored by Vietnamese consumers. According to WMS predictions, the compound annual growth rate of sausages and salami in Vietnam in the period 2020–2025 will reach 6.26% per year with a total market value estimated at 728.28 million USD by 2025 [4], and Vietnam already at number 23 (1,074.77 million USD) in 2022 [3]. This trend shows that sausage has great potential for development, so it attracted huge investments from food companies. Currently, there are many types of sausages from big brands available on the domestic market such as Vissan, CP, Dabaco, and Duc Viet. However, due to the popularity of these products, leading to the low price, many fake, low-quality, unlabelled, and unclear origin products are rampant, posing a threat to consumers' health [4, 5]. These products can contain nitrosamine and polycyclic aromatic hydrocarbon compounds that are carcinogenic and can cause colorectal adenoma [6, 7]. Some studies reported microbial contamination during sausage processing [8–11]. Although the samples were treated by heat, *Staphylococcus aureus, Escherichia coli,* and *Lactobacillus viridescens* were still detected, and some samples were beyond the limit for coliforms. To contribute to the quality control of sausages, grading measures are necessary.

Statistical methods have long been recognized as powerful tools in food classification and quality assessment [12]. Some studies reported that the utilization of statistical methods in chemistry tends to increase over the years. Among them, the principal component analysis (PCA) and cluster analysis (CA) are the two most favorable tools [13–15]. However, only statistical methods are insufficient for the assessment of the quality and identify food category; it is usually accompanied by the data obtained from measuring methods such as physicochemical property analysis (moisture, crude protein, ether-extractable fat, and ash) [16–20], meta-imaging spectra [21], microcomputing tomography [20, 22, 23], and chemical data (nutrient content and multielement content) [24, 25]. However, the determination of physicochemical properties in foods is time-consuming and complicated [16–20]. In the case of the utilization of hyperspectral imaging, additional algorithms should be used to reduce the influence of the distribution of meat and fat in the sausage [26]. Thus, to simplify the classification, spectral data from FT-IR analysis and multielemental composition from the ICP-MS technique were employed to conduct the multivariate statistical analysis.

Many studies have demonstrated the feasibility of this combination. Nesakumar et al. applied the combination of PCA and FT-IR to determine the moisture content and age of beetroot, with the accuracy of the beetroot quality prediction model up to 97.5% [27]. Yan Wang et al. [28] decided to combine PCA with ICP/MS/MS to explore each relationship between the content of 30 elements in rice and the production site. Based on the macronutrient content and cluster analysis, the samples were precisely divided into two main categories and six subcategories according to the place of production. Notably, there was a significant correlation between the elemental content in rice and the place of production, and thus, the origin of rice can be traced. In another study, the classification of pakchoi (*Brassica rapa L.* ssp*. Chinensis*) according to six growing regions using multivariate statistical methods (PCA) and ICP/MS has been applied successfully [29].

To the best of our knowledge, no studies that employed chemical analysis combined with multivariate statistics to identify sausages from different brands have been conducted. Therefore, in this paper, FT-IR and ICP-MS were the techniques combined with multivariate statistical methods to distinguish the brand names of sausage samples.

In the realm of food science and market dynamics, the necessity to distinguish between various sausage brands emerges from several pivotal concerns, paramount among which is the complexity and rapid expansion of the global and specifically Vietnamese sausage markets. This burgeoning diversity, while offering consumers a wide array of choices, concurrently engenders challenges in maintaining product authenticity and quality assurance. A critical aspect underpinning this study is the escalating incidence of adulteration within the market. The proliferation of sausage brands has been accompanied by an alarming rise in products that are not only counterfeit or of inferior quality but also potentially hazardous to consumer health. Such products frequently contain harmful substances such as nitrosamines and polycyclic aromatic hydrocarbons and are prone to microbial contamination, including pathogens such as Staphylococcus aureus, Escherichia coli, and Lactobacillus viridescens. These adulterated products not only undermine the integrity of established brands but also pose significant public health risks.

Consequently, there is an imperative need for robust and efficient methodologies capable of authenticating sausage brands to safeguard consumer interests and health. Traditional methods for quality assessment and brand differentiation in the sausage industry, while valuable, often fall short in terms of specificity, efficiency, and scalability. They are typically time-consuming, and labor-intensive, and may not provide the requisite resolution to distinguish between closely related or similarly formulated products. Addressing this lacuna, the present study introduces a novel approach that synergizes principal component analysis (PCA) with advanced chemical analysis techniques, namely, attenuated total reflectance-Fourier transform infrared (ATR-FTIR) spectroscopy and inductively coupled plasma-mass spectroscopy (ICP-MS). This methodological fusion is designed to exploit the unique chemical fingerprints of sausage samples, thereby enabling a more nuanced and precise differentiation of brands. The PCA, in particular, offers a powerful statistical tool to analyze complex datasets, reducing dimensionality and highlighting patterns and variations that are indicative of brand-specific characteristics.

Furthermore, the study's approach addresses the logistical and practical challenges associated with sausage quality control. By employing ATR-FTIR and ICP-MS, the research circumvents the limitations of more conventional physicochemical analysis methods. These advanced techniques allow for rapid, nondestructive analysis of the samples, providing a comprehensive profile of their chemical makeup, including both organic and inorganic constituents. The integration of PCA serves to distill this voluminous data into actionable insights, effectively differentiating between brands based on their distinct spectral and elemental signatures.

By establishing a reliable and efficient protocol for brand authentication, the research directly contributes to mitigating the risks associated with food adulteration. It provides a framework that can be adopted by regulatory bodies and manufacturers alike to ensure that the products reaching consumers are genuine, safe, and of the quality that they purport to be. In essence, this study not only addresses a specific market need but also contributes to the broader discourse on food safety, consumer protection, and the integrity of food supply chains.

## 2. Materials and Methods

### 2.1. Chemicals and Instruments

Nitric acid 65% (Merck, Darmstadt, Germany), hydrogen peroxide 30% (Merck, Darmstadt, Germany) solutions, and ultrapure deionized water with a resistivity of 18.2 MΩ cm were obtained from a Milli-Q Plus water purification system (Millipore, Bedford, MA, USA). The multielement standard solution consists of B, Na, Mg, Al, Si, K, Ca, V, Cr, Mn, Cu, Zn, Ga, As, Rb, Sr, Ag, Cd, Sb, Cs, Ba, Hg, and Pb (TraceCERT, Merck, Darmstadt, Germany), and nine rare earth elements (Sc, Y, La, Ce, Pr, Nd, Sm, Eu, and Gd) 10 mg/L each element were provided by Sigma-Aldrich (Missouri, USA).

The main instruments used include the ATR-FTIR spectrometer (Nicolet™ iS50, Thermo Fisher Scientific, USA), MARS6 microwave oven (CEM Corporation, Matthews, NC, USA), and iCap TQ ICP/MS (Thermo Fisher Scientific, Bremen, Germany).

### 2.2. Method

Operating parameters for ICP-MS measurement are shown in Table 1.

Statistical analysis of the data was performed using XLSTAT (Lumivero, Denver, USA) and STATISTICA 12 (Dell Software, USA). Principal component analysis (PCA) was applied to analyze data obtained from FT-IR and ICP-MS analyses to evaluate the distinctions between sausage samples from 6 brands. The outputs of the multivariate statistical analysis include a screening histogram to show the contribution of the principal components to the PCA model, a score scatter plot illustrating the separation of 6 different groups, a loading scatter plot explaining the influence of factors on clustering, and the moving range graph illustrating the means and the general distribution of the variables across the cases [29].

### 2.3. Sample Preparation

Sixty sausage samples were systematically obtained from six distinct brand names—Ebol, Lacus, Bucher, ĐNÁ, Le Gourmet, and Vissan—with each brand contributing 10 samples. These samples were collected from various supermarkets in Hanoi to ensure geographical diversity and thereby maximize the robustness of the study's findings.

#### 2.3.1. Sample Preparation

Each sausage sample, weighing approximately 50 grams, underwent a meticulous sample preparation procedure. Initially, the samples were minced and subsequently subjected to alcohol immersion for 15 minutes as a pretreatment step. This was followed by a drying process carried out at 70°C for 24 hours. Post-drying, the samples were ground to a fine consistency utilizing a Seka SK200 grinder. To ensure uniformity and eliminate moisture, the ground samples were enveloped in oil-absorbent pads and subjected to further grinding using a mortar and pestle. The homogenized samples were then stored at a temperature of 4°C until further analysis.

#### 2.3.2. Fourier Transform-Infrared (FT-IR) Spectroscopy Analysis

Prior to FT-IR analysis, the ground samples were sifted using sieves with mesh sizes ranging between no. 100 and no. 120, aimed at isolating particles with dimensions falling within the 0.125–0.150 mm range. This sieving process minimized the potential impact of radiation scattering attributable to variable particle sizes, thus ensuring analytical consistency. The FT-IR measurements were conducted using an ATR-FTIR instrument, covering a wavelength spectrum between 4000 and 400 cm^−1. Each sample was subjected to 128 scans with a spectral resolution of 4 cm^−1, and measurements were replicated six times to affirm the reliability of the data.

#### 2.3.3. Elemental Analysis

For the elemental composition determination, approximately 0.2 grams of each processed sample was weighed into a Teflon digestion tube. Subsequently, 4 mL of 65% nitric acid (HNO_3_) and 2 mL of 30% hydrogen peroxide (H_2_O_2_) were added. The tubes were allowed to stand overnight to maximize digestion efficiency. The following day, the samples were processed in a MARS6 microwave oven at a temperature setting of 190°C for a 30-minute digestion cycle. Upon reaching room temperature, the digested solutions were diluted using deionized water up to a 50 mL volume in a volumetric flask and then filtered through an 11 *μ*m filter paper before subsequent analytical procedures.

## 3. Results and Discussion

### 3.1. Results of the Spectral Signals and Elemental Composition of Sausage Samples

**Figure 1:**
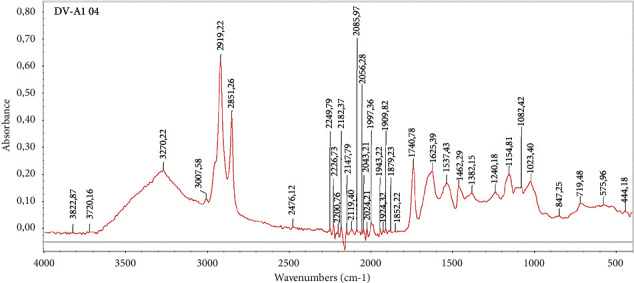
Main peaks of FT-IR spectrum of sausage samples.

Figure 1 depicts the characteristic FT-IR spectrum of the sausage sample with peaks concentrated in the fingerprint region. When superimposed on the obtained spectra, there is almost no difference between samples of different brands. In consideration of the full spectra of all samples, it was mostly impossible to differentiate the dissimilarities. Thus, peaks of the spectrum are selected to reduce unnecessary data. In which, the wavelength 3100–3700 cm^−1^ is the oscillation of the O-H functional group; the wavelength 2851–3007 cm^−1^ and 1380–1425 cm^−1^arise from the C-H vibrations while the bands at 1537–1625 cm^−1^ correspond to the C=C vibrations. The bands at 1023–1240 cm^−1^ result from the vibrations of group C–O. Since raw spectral data from FT-IR analysis are insufficient for the classification study, principle component analysis is the chosen multivariate statistical analysis to process the new data of selected peaks.

The FT-IR spectrum, predominantly concentrated in the fingerprint region as depicted in Figure 1, initially indicated minimal variance between samples of different brands, posing a challenge in distinguishing them based purely on raw spectral data. This observation underscores the complexity of the sausage matrix and suggests that the subtle differences in the composition of the sausages are not readily discernible in the fingerprint region of the FT-IR spectrum alone. The selection of specific peaks within the spectrum aimed at reducing data redundancy was a strategic approach. The wavelengths ranging from 3100 to 3700 cm−1, 2851–3007 cm−1, 1380–1425 cm−1, and 1023–1240 cm−1, corresponding to the vibrations of various functional groups (O-H, C-H, C=C, and C-O), provide crucial insights into the molecular structure of the sausages. However, these wavelengths alone were not sufficient to differentiate the brands effectively, indicating that the variances in the chemical composition of the sausages from different brands might be more nuanced and require a more refined analytical approach.

The utilization of principal component analysis (PCA) in this context emerges as a vital tool. PCA's capability to transform the original variables into a new set of uncorrelated variables, thereby reducing data dimensionality and enhancing the elucidation of data structure, is particularly useful. Through PCA, the study could reveal underlying patterns or trends in the spectral data that are indicative of brand-specific characteristics. These characteristics could arise from various factors such as differences in the quality and source of ingredients, processing techniques, and even the geographical origin of raw materials. In further discussions, it would be pertinent to delve into how these brand-specific characteristics, unraveled by PCA, could be linked to the particularities of each brand's production process, ingredient selection, and quality control measures. For instance, variations in the concentrations of certain elements such as sodium, potassium, or iron could be attributed to the use of different curing agents, spices, or meat types. The implications of these findings for future research are vast. They suggest that a combination of FT-IR spectroscopy and PCA, along with potentially other analytical techniques, could be employed more broadly in the food industry for quality control, authentication of origin, and even detection of adulteration.

In the detailed discussion of Table 2, which provides specific quantitative metrics for both essential and toxic elements in sausage samples from six different brands, certain numerical values stand out, highlighting the variation in elemental composition across these brands. Each brand's sample, represented by 10 individual samples and subjected to triplicate measurements, offers a robust statistical framework to analyze these elemental distributions.

Le Gourmet's samples, for instance, displayed remarkably low concentrations of elements such as aluminum (Al), chromium (Cr), manganese (Mn), iron (Fe), copper (Cu), and barium (Ba), with their levels falling below the instrument's limit of detection (LOD). This is particularly significant in the case of iron (Fe), an essential nutrient, where its insufficient presence in Le Gourmet sausages could raise questions about its nutritional adequacy.

In stark contrast, the Vissan brand demonstrated exceptionally high sodium (Na) levels, with an average concentration of 39,436.30 ± 4736.78 *μ*g/g. This concentration is notably high compared to standard nutritional recommendations and could have health implications, especially for individuals with hypertension or those on sodium-restricted diets. Such high sodium content in Vissan sausages underscores the importance of consumer awareness regarding dietary sodium intake.

Ebol brand sausages were characterized by their high magnesium (Mg) content, averaging 2,416.57 *μ*g/g. This contrasts sharply with the magnesium content in Le Gourmet sausages, which was only 446.03 *μ*g/g—less than half of that found in the Butcher brand, the next lowest, with an average magnesium concentration of 1,045 *μ*g/g. This significant difference in magnesium content among brands could be a reflection of the varying ingredient profiles or additives used.

Iron concentrations also varied considerably across brands. Vissan sausages contained a notably high iron concentration of 773.34 *μ*g/g, while Le Gourmet's iron levels were so low they fell below the LOD. This disparity in iron content could be a crucial factor for consumers making dietary choices based on iron intake requirements.

Mercury (Hg), quantified in *μ*g/kg due to its minimal occurrence, was another element analyzed. Although the presence of mercury in food is a concern for food safety, the concentrations in all sausage samples were below the FDA's reporting limit [23]. This finding, while reassuring, emphasizes the need for ongoing vigilance in monitoring toxic elements in food products.

These specific quantitative findings from the study reveal a diverse elemental landscape across different sausage brands. They highlight the necessity for consumers to consider the elemental composition of sausages in their dietary choices, especially given the potential health implications of elements such as sodium and iron. For manufacturers, these results underscore the importance of ingredient selection and processing methods in influencing the final product's elemental makeup, directly impacting the nutritional profile and safety of the sausages.

### 3.2. Data Analysis with Multivariate Analysis

Principal component analysis (PCA) serves as an invaluable computational strategy in multivariate statistics, primarily designed to reduce the high dimensionality inherent to complex datasets. By transforming the original variables into a new set of uncorrelated variables, termed principal components, PCA facilitates the elucidation of the data structure, revealing underlying patterns or trends that might otherwise go undetected.

Within the realm of food science, PCA has proven itself as a robust analytical method capable of classifying food products based on a myriad of physicochemical attributes. Recent advancements have extended its application to the categorization of food items based on their metal element profiles, serving as an indirect marker for geographical origin or processing techniques [30]. For example, a pivotal study by Singh and Ghosh [31] employed PCA in the classification of various honey brands, discovering that regional variations significantly influenced the metal content profiles, thereby allowing for effective differentiation [25]. Another compelling case is presented by Xu et al., who utilized PCA to segregate Chinese green tea samples, demonstrating that region-specific differences in metal element profiles could be clearly delineated using this approach [32].

The analytical power of PCA lies not just in dimensionality reduction but also in its ability to identify key variables—here, specific metal elements—that make substantial contributions to the variance within the data, thus becoming salient features for classification. For instance, in the context of quality and safety assessments of sausages, Ammor and Yaakoubi [33] leveraged spectral data in conjunction with PCA to differentiate strains of lactic acid bacteria in fermented sausages. Furthermore, PCA provides the added benefit of generating biplots or scatter plots, offering a visual representation that simplifies the interpretative process [32].

Considerable attention was devoted to the meticulous preprocessing and scaling of Fourier transform infrared spectroscopy (FTIR) spectral data for its incorporation into a principal component analysis (PCA) framework. The procedural steps were consistently implemented across all sections of the research to ensure methodological coherence. Specifically, the absorption peaks that were the focus of the multivariate analyses were carefully selected to correspond with distinct molecular vibrations relevant to the chemical composition of the samples under investigation.

The spectral regions that were targeted for this analysis include the following:

- Wavelengths ranging from 3100 to 3700 cm^−1^ and 1380 to 1425 cm^−1^, which are predominantly indicative of the vibrational modes associated with hydroxyl (O-H) functional groups
- Spectral bands spanning the wavenumber intervals of 2851 to 3007 cm^−1^ emanate from the vibrations of the C-H bonds commonly found in organic compounds
- Absorption peaks were observed between 1537 and 1625 cm^−1^, representing C=C vibrational modes
- Bands ranging from 1023 to 1240 cm^−1^ are associated with the C-O functional group vibrations.

Following the objectives and requirements of the study, no logarithmic transformations were applied to the spectral data. The rationale behind this decision was to maintain methodological alignment across different sections of the study. Rather, a process of numerical conversion and scaling was meticulously executed to ensure that each variable contributed uniformly to the PCA model. For this purpose, the raw spectral data were processed using The Unscrambler X software (CAMO, Oslo, Norway). This specialized software facilitated the transformation of the spectral data into a numerical matrix format conducive to subsequent multivariate statistical analyses.

**Figure 2:**
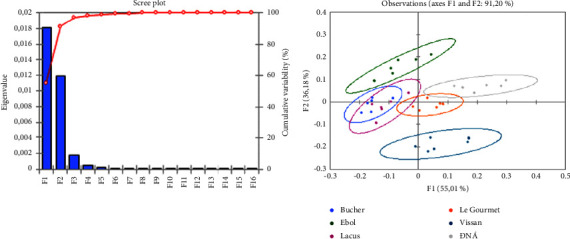
Scree plot and PCA visualization of the sausage dataset obtained by FT-IR.

In this study, Figure 2 shows the results of PCA performance on the FT-IR dataset of 6 sausage brands. It can be seen that the first 2 PCs account for over 90% of the total sample variation. Therefore, it can be said that these PCs carry the most significant information of the dataset and the PCA visualization on the new coordination was reliable. The separation of 6 sample groups corresponding to their brands can be seen on the PCA score plot. Notably, sausages from 4 brands of Vissan, ĐNÁ, Le Gourmet, and Ebol were the most classified samples due to their distinctive distributions on the PCA plot. Although the distribution of a few samples from Lacus and Le Gourmet nearly overlapped, in general, the sausages of these two brands are still distinguishable. On the other hand, it is noticeable that the spectral signals of the samples from Bucher and Lacus are indistinctive. Most of the samples of these two brands are overlapped and unseparated. Girardeau and Passot [34] used PCA to analyze the fluorescence spectra of lactic acid bacteria. The results show similarities to this experiment's results as the discriminant map factors show certain factors are identified while several appear overlapped. The combination of the infrared spectroscopy dataset and the multivariate statistical method has shown the feasibility of classifying sausage brands.

**Figure 3:**
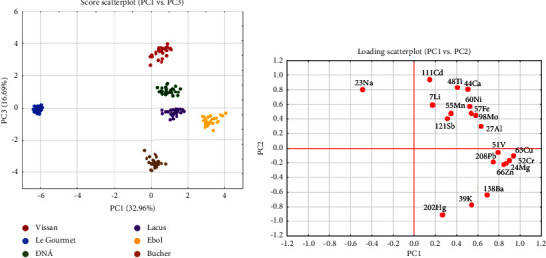
PCA scores scatterplot of principal components 1 and 3, and PCA loading scatterplots.

Another dataset of chemical composition commonly used in food discrimination is the metal content [35, 36]. The average metal content of 60 sausage samples was determined by the ICP-MS instrument (Table 2). The average concentration of 20 elements was selected as the input data for the PCA analysis, employing STATISTICA software. The results of the PCA analysis are presented in Figures 3(a) and 3(b). In the two-way point scatter plot based on PC1 and PC3 (Figure 3), the sausage samples from the 6 brands were separated. This highlighted the ability of the PCA method to distinguish the brand name origin of the sausage samples based on the variation of elemental composition. Additionally, the degree of variables' (elemental) contribution to the separation of sample classes and the correlation among variables is depicted in the loading scatterplot (Figure 3(b)). There are 6 variables that play the principal role in the separation of each class, including Cu, Cr, Mg, Zn, Cd, and Hg. Notably, the first four elements are positively correlated and carry the highest weight in the classification of the first PC. Meanwhile, although Cd and Hg are also the major contributors to the distribution of 6 sample classes on PC2, they display a negative correlation due to their opposite values nearly 1 and -1 on PC2, respectively.

**Figure 4:**
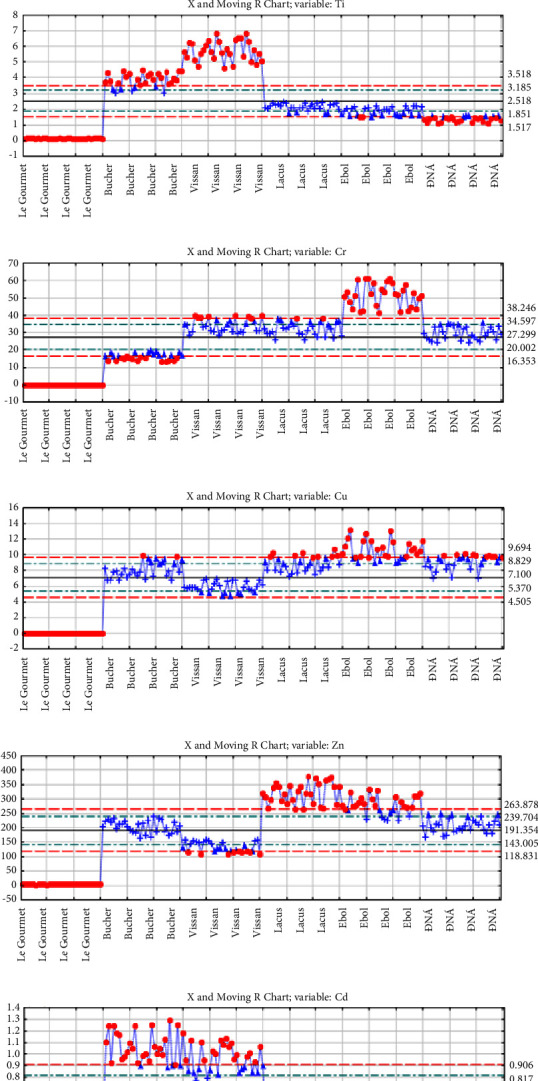
Moving range chart of (a) Mg, (b) K, (c) Ca, (d) Ti, (e) Cr, (f) Cu, (g) Zn, (h) Cd, (i) Ba, and (j) Hg. The vertical axis illustrates the content (*μ*g/g, dried weight base) of each element in the sample. The horizontal axis shows the names of the areas.

The moving range diagrams illustrate the means and the common distributions of the variables in the cases. The Ebol sample had a much higher concentration of Mg, Cr, and Cu than the rest of the samples, which can be used as a distinguishing mark from other brands (Figure 4(a)). Meanwhile, Vissan models had significantly higher Ti content than other brands. The combination of Ca and Cd factors was identified as the main distinguishing factor for sausages produced by Bucher. The combination of K and Zn factors also contributed to the pattern differentiation of Lacus. As for the Le Gourmet sample, no elemental isotopes were identified with high concentrations; however, it can be distinguished based on the Hg content which is nearly twice as high as that of the Bucher and Vissan samples. Similarly, it was difficult to determine the factors that distinguish sausages from the ĐNÁ brand, except that the Hg content was slightly higher than that of the other brands.

Multivariate statistics serves as an indispensable tool in the realm of complex data analysis, particularly when dealing with multidimensional variables that require simultaneous examination to extract meaningful patterns or trends. The advantage of multivariate methods such as principal component analysis (PCA) or cluster analysis is their ability to distill multiple variables into essential factors, thereby aiding in more incisive interpretation and decision-making.

However, the size of the data set introduces its own set of challenges that cannot be overlooked. Large and complex data sets demand considerable computational power and memory resources, which can be a limiting factor especially if the analyses are being run on standard computing devices. This computational burden not only affects the speed but can also influence the accuracy and reliability of the results, especially when computational shortcuts or approximations are employed to manage large data sets.

Therefore, preparatory measures are often required to enable efficient and accurate analyses. Training in advanced computational methods and statistical software can offer researchers the skills necessary to optimize data analysis procedures. For instance, knowledge of high-performance computing practices can enable the execution of analyses that would otherwise be computationally prohibitive.

Additionally, the use of secondary algorithms aimed at variable reduction can be invaluable. In contexts like spectral analysis, where hundreds or even thousands of variables might be collected for each sample, algorithms can be employed to eliminate redundant or irrelevant variables, thus reducing the dimensionality of the data. This simplification not only eases the computational burden but also helps in avoiding overfitting, thereby improving the model's predictive accuracy. The use of powerful computing devices equipped with high-speed processors and ample memory can substantially mitigate the challenges posed by large data sets. In some cases, cloud-based computing solutions or parallel processing techniques can be employed to distribute the computational load across multiple devices or servers, making it feasible to analyze extremely large or complex data sets.

In the multivariate analysis, the application of PCA in this research provides a quintessential example of its utility in handling high-dimensional and complex datasets. The PCA methodology successfully transformed the spectral and elemental data into a set of principal components, offering a more insightful interpretation of the data. This approach has revealed subtle but significant differences in the chemical profiles of the six sausage brands, which were not immediately apparent in the raw data. The PCA's ability to distill these nuances is particularly evident in the differentiation of the brands based on their metal element profiles. The specific elements, including Cu, Cr, Mg, Zn, Cd, and Hg, emerged as key differentiators among the brands. This highlights PCA's capability in not just reducing the dimensionality of the dataset but also in pinpointing specific variables that are critical in distinguishing between the sausage brands. The correlation observed among these elements, as well as their varying concentrations in different brands, suggests a complex interplay of factors that could be influenced by the brand's source of ingredients, processing methods, and quality control protocols. For instance, the significantly higher concentrations of Mg, Cr, and Cu in Ebol samples, compared to other brands, could be indicative of specific processing practices or ingredient sources unique to Ebol. Similarly, the high Ti content in Vissan and the distinct combination of Ca and Cd in Bucher sausages offer a unique fingerprint of these brands. Such elemental disparities provide valuable insights into the potential health implications of these products, especially considering elements such as sodium and mercury, which have direct relevance to consumer health.

This study's findings underscore the complexity of food matrices like sausages and the challenges in their analysis. The successful application of PCA in this context demonstrates the method's potential in food science research, particularly for brand differentiation and quality control. It also suggests avenues for future research, such as exploring the underlying reasons behind these elemental variations and their implications for food safety and nutrition. However, the analysis also brings to light the computational challenges inherent in handling large datasets. The need for powerful computing resources and sophisticated statistical software is evident. This requirement underscores the importance of investing in advanced computational infrastructure and training for researchers to effectively manage and interpret complex datasets. Such capabilities are essential for harnessing the full potential of multivariate statistical methods like PCA, ensuring not only the efficiency of the analysis but also the accuracy and reliability of the findings.

In summary, this research, through its innovative use of PCA in conjunction with FT-IR and ICP-MS analyses, offers a novel approach to food product differentiation and quality assessment. The study contributes significantly to the field of food science, providing a template for future research in food product authentication and safety evaluation. It also highlights the need for continued advancements in computational methods and resources to keep pace with the increasing complexity of food science research.

## 4. Conclusion

Through the FT-IR method, the study demonstrated the feasibility of using infrared spectroscopy to classify and distinguish between different sausage brands. This not only bolsters FT-IR spectroscopy as a promising tool for food differentiation but also emphasizes its sensitivity to the variations in the composition of such products. Results from ICP-MS provided a clear distinction between sausage brands based on their elemental composition. Notably, elements such as K, Ca, Ti, Ba, and Ni were pivotal in driving these distinctions. This sheds light on the importance of considering elemental compositions when characterizing and classifying food products.

This study underscored the effectiveness of combined analytical methodologies in differentiating commercially produced sausages. By integrating FT-IR spectroscopy and ICP-MS metal analysis, a robust platform for distinguishing between sausage brands based on compositional nuances was developed. Specifically, the infrared spectroscopy indicated potential in food differentiation due to its sensitivity to composition variations. Concurrently, the elemental composition results from ICP-MS, emphasizing the role of elements such as K, Ca, Ti, Ba, and Ni, distinctly classified sausage brands. Through PCA's application to both datasets, the research illuminated the complementary nature of these sources in food product classification. Ultimately, our findings highlight a promising avenue for enhancing food product characterization, a critical step in ensuring food safety, authenticity, and quality.

## Figures and Tables

**Table 1:** Operating parameters of ICP-MS.

Parameter	Setting
RF power	1400 W
Plasma gas flow rate	15 L/min
Carrier gas flow rate	1.05 L/min
Makeup gas flow rate	0.9 L/min
Spray chamber temperature	2°C
Points per spectral peak	3
Sweeps per reading	10
Measured isotopic positions of elements	^11^B, ^23^Na, ^24^Mg, ^27^Al, ^28^Si, ^39^K, ^43^Ca, ^51^V, ^52^Cr, ^55^Mn, ^63^Cu, ^66^Zn, ^69^Ga, ^75^As, ^85^Rb, ^88^Sr, ^107^Ag, ^111^Cd, ^121^Sb, ^133^Cs, ^137^Ba, ^202^Hg, and ^208^Pb

**Table 2:** Average concentration of the 20 elements in sausages from 6 brands (*μ*g/g, dried weight) (*n* = 3).

	Le Gourmet	Bucher	Vissan	Lacus	Ebol	ĐNÁ
Li	0.40 ± 0.05	0.73 ± 0.08	0.50 ± 0.05	0.51 ± 0.06	0.39 ± 0.04	0.55 ± 0.07
Na	29,148.65 ± 3085.08	29,759.96 ± 3641.09	39,436.30 ± 4736.78	5,140.04 ± 569.46	4,255.23 ± 384.79	3,650.74 ± 502.5
Mg	446.03 ± 57.46	1,045.00 ± 139.34	1,953.71 ± 230.75	2,198.63 ± 200.07	2,416.57 ± 304.6	1,703.86 ± 211.07
Al	<LOD^*∗*^	418.36 ± 44.41	112.12 ± 13.23	211.63 ± 21.07	301.54 ± 30.33	146.52 ± 15.00
K	13,959.00 ± 1,798.28	9,294.20 ± 1094.10	13,668.10 ± 1563.76	31,220.18 ± 3866.97	31,018.45 ± 3,421.01	25,327.77 ± 2,760.52
Ca	59.87 ± 6.84	733.63 ± 70.45	657.54 ± 81.21	394.81 ± 50.19	373.36 ± 43.37	255.00 ± 30.66
Ti	0.14 ± 0.02	3.85 ± 0.45	5.73 ± 0.64	2.14 ± 0.24	1.87 ± 0.25	1.38 ± 0.16
V	0.49 ± 0.06	0.69 ± 0.08	0.70 ± 0.08	0.65 ± 0.07	0.85 ± 0.10	0.76 ± 0.07
Cr	<LOD^*∗*^	16.23 ± 1.81	34.18 ± 3.91	32.25 ± 3.98	51.43 ± 6.6	29.71 ± 3.83
Mn	<LOD^*∗*^	4.73 ± 0.58	31.24 ± 3.29	4.16 ± 0.56	8.09 ± 0.99	14.43 ± 1.90
Fe	<LOD^*∗*^	214.85 ± 24.7	773.34 ± 96.94	285.15 ± 32.58	341.73 ± 38.99	289.54 ± 31.34
Ni	3.8 ± 0.46	70.28 ± 8.23	31.75 ± 3.72	28.38 ± 3.00	43.41 ± 4.74	15.76 ± 1.79
Cu	<LOD^*∗*^	8.11 ± 1.02	5.87 ± 0.65	8.94 ± 0.98	10.59 ± 1.26	9.10 ± 0.94
Zn	4.45 ± 0.47	204.21 ± 21.16	133.04 ± 17.33	320.95 ± 36.36	277.29 ± 31.57	208.18 ± 23.81
Mo	0.44 ± 0.05	0.76 ± 0.08	1.14 ± 0.11	0.73 ± 0.08	0.75 ± 0.09	0.92 ± 0.10
Cd	0.41 ± 0.05	1.06 ± 0.13	0.95 ± 0.11	0.46 ± 0.06	0.46 ± 0.05	0.49 ± 0.06
Sb	0.12 ± 0.02	0.24 ± 0.03	0.14 ± 0.02	0.12 ± 0.02	0.21 ± 0.03	0.10 ± 0.01
Ba	<LOD^*∗*^	1.40 ± 0.15	5.11 ± 0.59	32.44 ± 3.65	43.50 ± 4.63	17.83 ± 1.86
Hg (**μ**g/kg)	0.56 ± 0.06	0.36 ± 0.04	0.35 ± 0.04	0.74 ± 0.08	0.76 ± 0.08	0.77 ± 0.09
Pb	0.71 ± 0.08	4.64 ± 0.51	4.14 ± 0.46	4.40 ± 0.51	6.37 ± 0.53	8.95 ± 0.96

^*∗*^LOD: limit of detection (3x standard deviation = 0.003 **μ**g/kg). Mean concentrations of elements in sausage samples (**μ**g/g) from six brands. Values represent the mean ± standard deviation of triplicate measurements for each of the 10 samples per brand. Triplicate measurements were conducted to ensure analytical robustness.

## Data Availability

The data used to support the findings of this study are included in the article.
